# A new borne with very complex aortic anatomy: diagnosis and treatment challenge—case report

**DOI:** 10.1186/s43044-020-00086-w

**Published:** 2020-08-20

**Authors:** Amr Mansour

**Affiliations:** Congenital and Structural Heart Disease Unit, Cardiology Department, Ain Shams University, Cairo, Egypt

**Keywords:** Critically ill neonate, Aortic interruption, Aortic coarctation, Interposition graft, Case report

## Abstract

**Background:**

Interrupted aortic arch (IAA) is a congenital malformation of the aortic arch which involves 3 out of 1 million live births. This congenital anomaly rarely occurs as an isolated lesion and is often associated with other intracardiac malformations, most commonly ventricular septal defect and patent ductus arteriosus (PDA). The diagnosis and surgical treatment of aortic interruption is usually challenging and may require multiple operations throughout the patient’s life.

**Case presentation:**

This case represents a neonate with interrupted aortic arch (type B) and a very long segment of descending aorta hypoplasia and complex anatomy. The patient escaped early diagnosis at birth and presented few days later by a picture that mimicked severe sepsis and shock.

His aortic anatomy was very complex and he was treated with long extra-anatomical aortic interposition graft.

**Conclusion:**

Aortic interruption is a rare congenital anomaly and is considered an extreme form of aortic coarctation.

It sometimes escapes early diagnosis due to the presence of patent ductus arteriosus and present later with shock and lactic acidosis. Sometimes the aortic anatomy is very complex and requires unusual surgical techniques for its repair.

## Background

Interrupted aortic arch (IAA) is a rare congenital anomaly, and it is considered an extreme form of aortic coarctation. Celoria and Patton classified IAA into three major types [1, 5].

If not taken in account with high degree of suspicion, the patient may not be diagnosed at birth with few patients may even escape diagnosis till adulthood [6]. Although there are standard surgical techniques for the repair of IAA, sometimes the aortic anatomy is very complex requiring multiple operations and unusual treatment strategies [2, 3].

This case represents a neonate with interrupted aortic arch (type B), very long segment of descending aorta hypoplasia, and complex anatomy. He escaped diagnosis at birth and presented few days later by a picture that mimics severe sepsis and shock. He was treated with long extra-anatomical aortic interposition graft.

## Case presentation

A full-term, newly born first baby for a non-consanguineous parent, presented 6 days after delivery with severe tachypnea, irritability, poor suckling, poor feeding, and lethargy.

The antenatal period was uneventful with no maternal illness or exposures, and the delivery was vaginal and was uneventful too.

On examination, the patient showed no dysmorphic feature, and he was in poor general condition, feeble crying, with marked tachypnea and tachycardia.

His arterial blood pressure was 64/30 mmHg, mean pressure 41 mmHg as measured from his left arm, heart rate of 163 bpm. The arterial blood pressure in his right arm was undetectable.

His skin was mottled with grayish appearance, and there was no peripheral pulsation in the lower half of his body. He had delayed capillary refilling (more than 6 s). Cardiac examination revealed a grade 2 systolic murmur on the base of the heart, with accentuated second heart sound.

His blood gases revealed lactic acidosis, hyperkalemia with high anion gap, and normal blood glucose level.

Laboratory investigations including blood cultures were withdrawn immediately, the patient was admitted to the neonatal intensive care unit (ICU), a central line was inserted, and the patient was intubated. Supportive treatment was started.

### Differential diagnosis

Neonatal sepsis was the first differential diagnosis with septic shock, and other differential diagnoses are severe aortic coarcatation with duct-dependent systemic circulation and aortic interruption.

### Echocardiography data

Situs solitus, levocardia, with normal connections, normal biventricular sizes and systolic functions, normal left-sided cardiac valves appearances with mild mitral regurgitation, with tricuspid aortic valve showing trivial aortic regurgitation. There was moderate degree of tricuspid regurgitation, and the estimated pulmonary artery pressure was found to be 65 mmHg after calculation of the right atrial pressure from the inferior venae cava size and variation with respiration.

There was a small mid-muscular ventricular septal defect (VSD) measuring 4 mm shunting bidirectionally with stretched foramen ovale.

There was a failure of clear visualization of the aortic arch and descending aorta from the supra-sternal window. The abdominal aortic flow from the subcostal views was non-pulsatile flow (monophasic flow) with minimal flow across a small closing patent ductus arteriosus (PDA).

### Multidetector computed tomography (MDCT)

Right-sided aortic arch with mirror image arrangement of the arch vessels, aortic arch interruption after the right common carotid (type B aortic interruption), the proximal segment of the descending aorta after the interruption is markedly hypoplastic, under-filled, and receives its blood supply via multiple tiny collaterals with small closing PDA supplying the aorta distal to the interrupted segment.

The right subclavian artery arises from the descending aorta distal to the interrupted segment.

The length of the interrupted segment was about 10 mm. The proximal 20 mm of the descending thoracic aorta distal to the interruption was hypoplastic, under-filled, and measuring about 2 mm in diameter; the aorta at the diaphragm measured 5.8 mm.

**Fig. 1:**
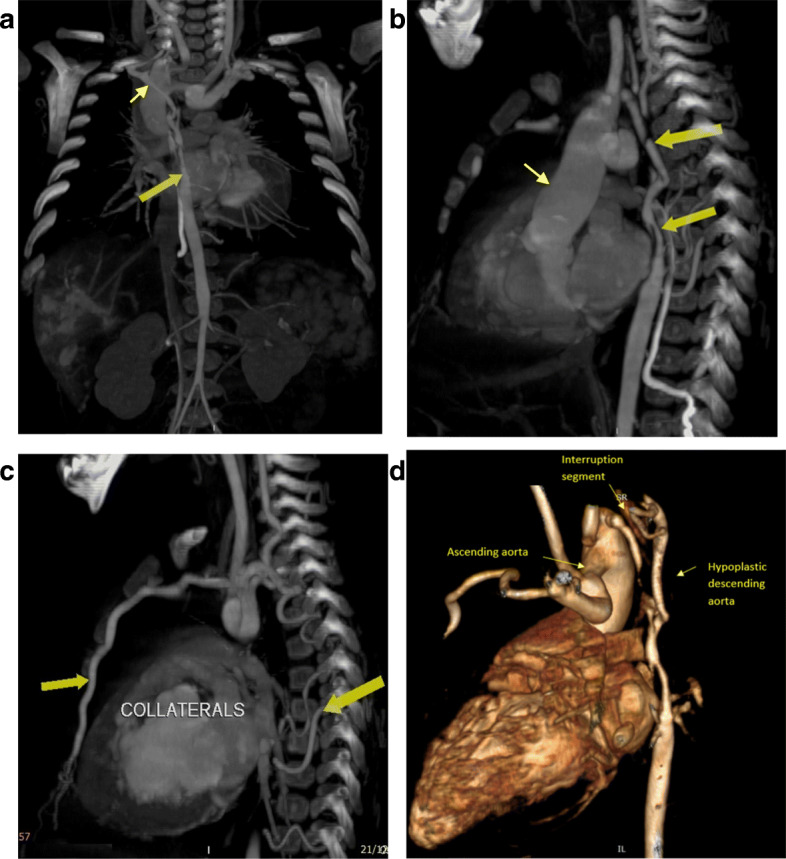
**a** MDCT, multi-planar reconstruction (MPR) image with maximal intensity projection (MIP) showing a long segment of descending aorta hypoplasia distal to the interruption (large yellow arrow), small right subclavian artery ( small yellow arrow) coronal view. **b** MDCT, multi-planar reconstruction (MPR) image with maximal intensity projection (MIP) showing ascending aorta (small yellow arrow), and the long segment of descending aorta hypoplasia distal to the interruption (large yellow arrow), sagittal view. **c** MDCT, multi-planar reconstruction (MPR) image with maximal intensity projection (MIP) showing the anterior and posterior dilated collaterals (large yellow arrows), sagittal view. **d** 3D MDCT volume rendering image shows the interruption segment and long segment of aortic hypoplasia

**Fig. 2:**
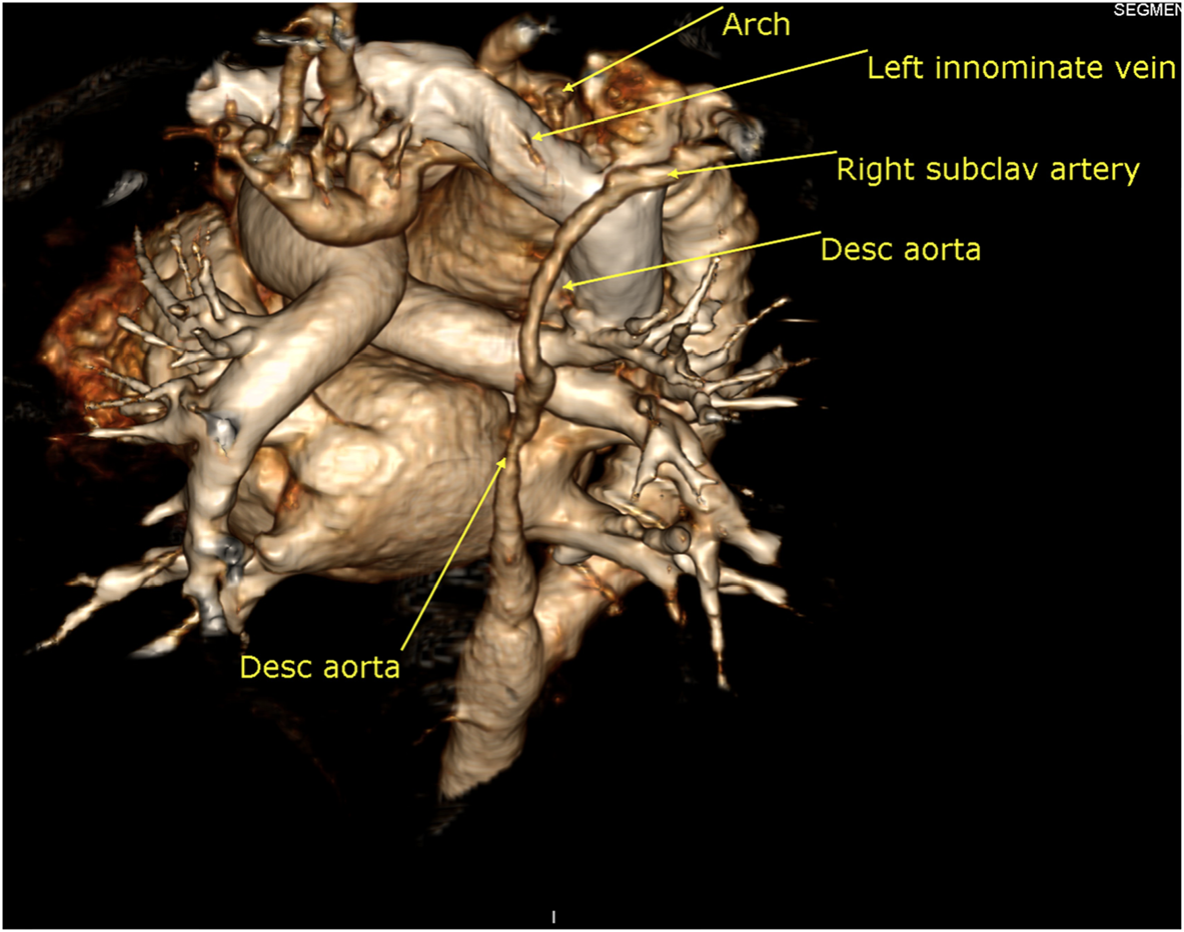
3D MDCT volume rendering image shows the interruption segment and long segment of aortic hypoplasia

The lower half of the body receives blood supply via anterior and posterior collateral circulations between the arch vessel branches (except the right subclavian artery) and the descending aorta and its branches and via a small PDA (Figs. 1 and 2)

### Chromosomal testes

The presence of chromosome 22q11 deletion was excluded. Intravenous prostaglandin was started immediately with supportive treatment. The patient was referred for the cardiothoracic surgery.

The surgery was done after the patient’s stabilization late in the neonatal period in another cardiac center through a median sternotomy and selective cerebral perfusion by an extra-anatomical aortic interposition graft due to the relatively long segment of interruption, the small diameter of the descending thoracic aorta distal to the interrupted segment, and complex anatomy that precluded extended end to end anastomosis and primary repair.

### Follow-up

**Fig. 3:**
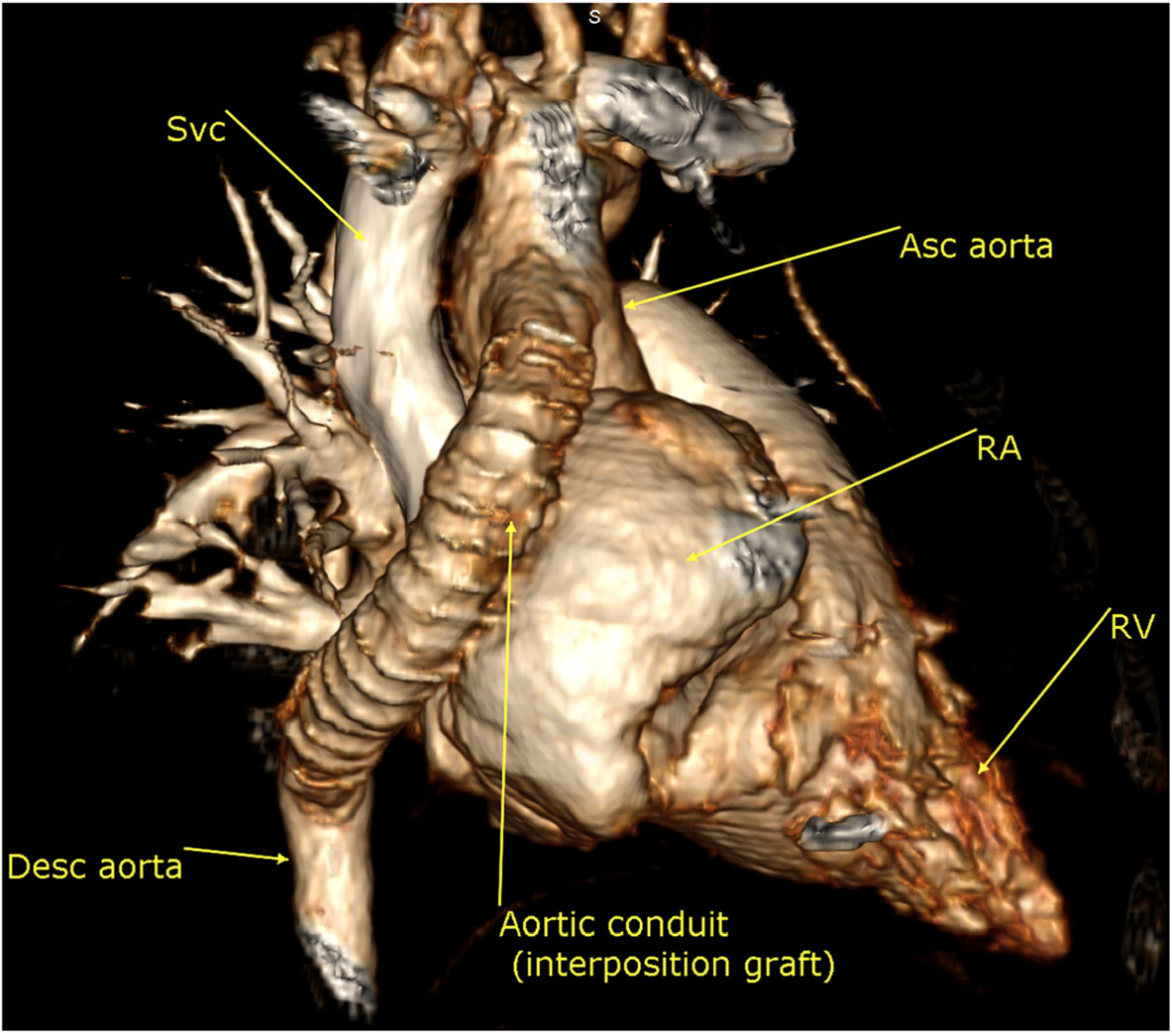
3D volume rendering image showing the aortic inter-position graft from the right lateral aspect

**Fig. 4:**
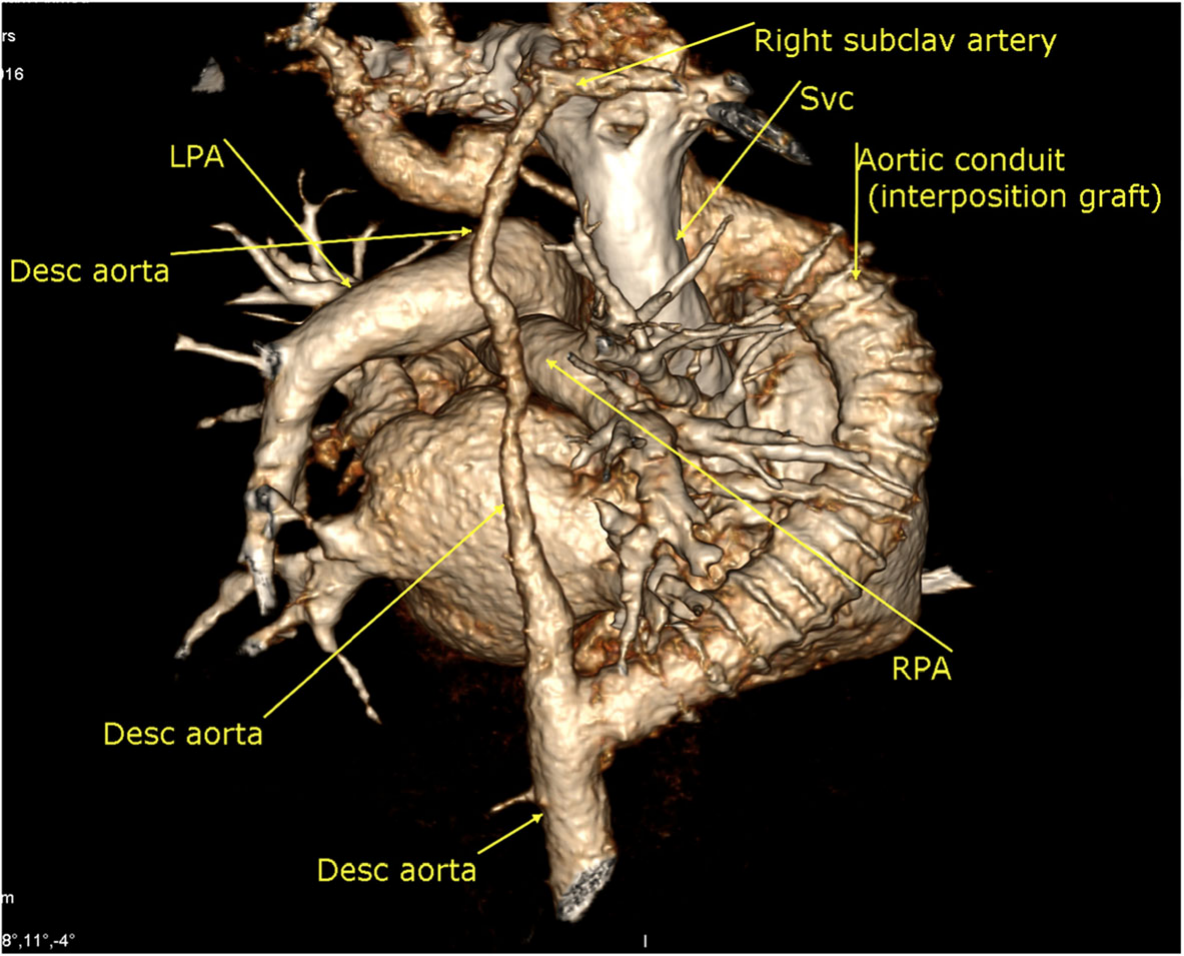
3D volume rendering image showing the aortic inter-position graft and the long segment of descending aorta hypoplasia from the posterior aspect

One year after surgical repair MDCT showed a patent conduit (extra-anatomical aortic inter-position graft) from the ascending aorta that runs on the right lateral aspect of the heart, then its turns posteriorly to join the descending thoracic aorta just above the level of the diaphragm; there was no significant stenosis within. It measures about 10 × 10 mm in diameter, 70–75 mm in length (Figs. 3 and 4).

## Discussion

Aortic interruption is a relatively uncommon congenital heart disease accounting for 1.5% of all congenital heart disease [1], with type B being the most common form accounting for (52–90%) [2, 3].

It is estimated that about 68% of the patient has associated chromosomal deletion and about 50% of patients have DiGeorge syndrome (abnormal facies, congenital heart defects, hypoparathyroidism, cognitive, behavioral and psychiatric problems, increased susceptibility to infections due to thymic aplasia, or hypoplasia) [4].

Our case did not have fetal echocardiography and was not diagnosed at birth mostly due to large PDA supplying the descending aorta; it was presented few days later by picture of shock, lactic acidosis, and hypoperfusion that poses some challenges in diagnosis.

There are different surgical techniques that are used for treatment of interrupted aortic arch, and some patients require multi-staged operations through their lifetime [1–3].

In our case, the surgeon could not do primary repair apparently due to the complex anatomy, so an extra-anatomical interposition graft was placed.

## Conclusion

Aortic interruption may escape diagnosis at birth and present later in a picture that may be confusing with septic shock and lactic acidosis, high index of suspicion is mandatory for diagnosis. Rarely, aortic anatomy is very complex and needs unusual surgical techniques for repair.

## Data Availability

The datasets used and/or analyzed during the current study are available from the corresponding author on reasonable request.

## Abbreviations

IAA: Interrupted aortic arch
ICU: Intensive care unit
VSD: Ventricular septal defect
PDA: Patent ductus arteriosus
MDCT: Multi-detector computed tomography
3D: Three-dimensional
MPR: Multi-planar reconstruction
MIP: Maximal intensity projection

## References

[1] Luciani G.B., Ackerman R.J., Chang A.C., Wells W.J., Starnes V.A: One-stage repair of interrupted aortic arch, ventricular septal defect, and subaortic obstruction in the neonate: a novel approach, J Thorac Cardiovasc Surg , 1996, vol. 111 (pg. 48-358) 10.1016/s0022-5223(96)70444-0 8583808

[2] Tláskal T., Hucin B., Kucera V., Vojtovic P., Gebauer R., Chaloupecky V., Skovranek J.: Repair of persistent truncus arteriosus with interrupted aortic arch, Eur J Cardiothorac Surg , 2005, vol. 28 November (5)(pg. 736-741) 10.1016/j.ejcts.2005.08.014 16194613

[3] Pankaj Kumar Mishra, Management strategies for interrupted aortic arch with associated anomalies, Eu J Cardio-Thoracic Surgery, Volume 35, Issue 4, April 2009, Pages 569–576, 10.1016/j.ejcts.2008.12.044. 10.1016/j.ejcts.2008.12.044 19233679

[4] Van Mierop L.H., Kutsche L.M.. Cardiovascular anomalies in DiGeorge syndrome and importance of neural crest as a possible pathogenetic factor, Am J Cardiol , 1986, vol. 58 (pg. 133-137) 10.1016/0002-9149(86)90256-0 3728313

[5] Celoria GC, Patton RB (1959). Congenital absence of the aortic arch. Am Heart J.

[6] Shirani S, Soleymanzadeh M (2013). Diagnosis of aortic interruption by CT angiography. Pol J Radiol.

